# Comparison of Color Light-Emitting Diode Corneal Topographer and Dual Rotating Scheimpflug–Placido Topographer

**DOI:** 10.1155/2018/6764805

**Published:** 2018-12-27

**Authors:** Jae Hyuck Lee, Yong Woo Lee, Jong Soo Lee, Michael C. Knorz, Gerd U. Auffarth, Chul Young Choi

**Affiliations:** ^1^Department of Ophthalmology, Kangbuk Samsung Hospital, Sungkyunkwan University School of Medicine, Seoul, Republic of Korea; ^2^Department of Ophthalmology, Pusan National University Hospital, Pusan National University School of Medicine and Medical Research Institute, Yangsan, Republic of Korea; ^3^Medical Faculty Mannheim of the University of Heidelberg, Mannheim, Germany; ^4^International Vision Correction Research Centre (IVCRC), David J. Apple International Laboratory on Ocular Pathology, Department of Ophthalmology, University of Heidelberg, Heidelberg, Germany

## Abstract

**Purpose:**

To compare measurements of anterior and posterior keratometric values, using a color light-emitting diode corneal topographer and a dual rotating Scheimpflug–Placido topographer.

**Methods:**

Anterior and posterior corneal index measurements were performed using a color light-emitting diode corneal topographer (Cassini) and a dual rotating Scheimpflug–Placido topographer (Galilei G4) and then compared. The paired *t*-test, intraclass correlation coefficients (ICCs), and Bland–Altman plots were used to evaluate the agreement between measurements.

**Results:**

Sixty postrefractive surgery eyes and 60 normal eyes were evaluated. Both the color light-emitting diode corneal topographer and the dual rotating Scheimpflug–Placido topographer provided highly repeatable corneal measurements (ICC > 0.969). The agreement levels between the 2 devices for anterior corneal power, astigmatism magnitude, and *J*0 and *J*45 values were ICC > 0.906 for the total group. However, the ICC values for posterior corneal power, astigmatism magnitude, and *J*0 and *J*45 values were lower than 0.681 for the total group.

**Conclusions:**

The anterior keratometric values obtained by the color light-emitting diode corneal topographer and the dual rotating Scheimpflug–Placido topographer showed high agreement levels, but the posterior keratometric values showed lower agreement levels.

## 1. Background

Accurate evaluations of corneal biometry measurements are important in cataract and refractive surgery [1]. As any error in corneal power calculation can directly lead to postoperative refractive surprises, the instruments used for keratometric measurements are crucial [2].

Many devices for corneal power estimation are available nowadays, and the dual rotating Scheimpflug–Placido topographer (Ziemer Ophthalmic Systems AG) is a hybrid device that combines dual rotating Scheimpflug cameras with a Placido disk. It is a widely used instrument because it can provide not only anterior and posterior corneal power measurements but also corneal curvature, pachymetry, and maps.

The Cassini (i-Optics Corp. v2.4.1) was recently released as the first commercially available point-source color light-emitting diode (LED) topographer that obtains a corneal image based on the reflection of individual points of light [3]. Although several studies have compared the color-LED corneal topographer with other devices, few studies have compared it with the dual rotating Scheimpflug–Placido topographer, and no studies to date have considered posterior corneal evaluation. Our purpose in this study was to compare the measurements of anterior and posterior corneal indices taken by the color-LED corneal topographer and the dual rotating Scheimpflug–Placido topographer.

## 2. Methods

This prospective cross-sectional study examined healthy subjects from the Department of Ophthalmology, Kangbuk Samsung Hospital, Seoul, South Korea. The study adhered to the tenets of the Declaration of Helsinki and was approved by the Institutional Review Board of Kangbuk Samsung Hospital. Written informed consent was obtained from each subject before inclusion in the study. In this study, we followed the methods of Lee et al. [4].

Inclusion criteria were healthy individuals aged 18 to 40 years with a spherical equivalent ranging from +1.00 to −6.00 diopters (*D*) to rule out high myopia which can cause extreme refractive errors. The best-corrected visual acuity was 0.00 logMAR in all eyes. Exclusion criteria included a history of ocular pathology, ocular trauma, contact lens wear, pregnancy, systemic or local medications, and ocular surgeries other than laser refractive surgery for myopia. In the postrefractive surgery group, we only included patients who underwent surgery more than a year ago and who did not have any subjective visual acuity change, discomfort, or history of ophthalmic treatment. In addition, patients with astigmatism of more than 2.50*D*, *K* values higher than 47.2*D* in any axis or inferior-superior (I-S) values (differences in I-S keratometry) more than 1.4*D* were excluded from this study to rule out keratoconus patients [5].

One eye from each subject was used for statistical analysis. Eyes were divided into 2 groups according to their history of myopic laser refractive surgery. The refractive group consisted of eyes with previous refractive surgery, while the normal group consisted of eyes that had not received refractive surgery.

### 2.1. Repeatability

Twenty participants (20 eyes) were included in the assessment of device repeatability, and 10 eyes had a history of refractive surgery for myopia. Three consecutive measurements were performed, and intraclass correlation coefficients (ICCs) were calculated for each measurement for anterior and posterior *K* and astigmatism values. The ICC is the ratio of between-subjects variance to the sum of the pooled within-subject variance and between-subjects variance. It expresses the consistency of repeated measurements and ranges from 0 to 1. An ICC smaller than 0.75 indicates poor repeatability, from 0.75 to 0.89 represents moderate repeatability, and greater than 0.90 corresponds to high repeatability [6]. All measurements in this study were performed by the same person experienced in ophthalmic examinations.

## 3. Measurements

All eyes were measured using both a dual rotating Scheimpflug–Placido corneal topographer and a color-LED corneal topographer.  Table 1 shows the characteristics and measurement settings used for each system. All measurements were performed continuously in individual subject between 10 a.m. and 1 p.m. to avoid the effects of diurnal variation in corneal indices [7].

### 3.1. Dual Rotating Scheimpflug–Placido System

The Galilei G4 uses 2 cameras in opposite positions in combination with a Placido disk with 20 Placido rings to analyze the shape of the cornea. Double rotating systems prevent and compensate for errors with oblique angle imaging. By detecting the edge in the dual-Scheimpflug images, the shape of the posterior cornea can be assessed. The total acquisition time was approximately 0.75 seconds, and more than 122,000 points were scanned. The simulated *K* (sim*K*) values were calculated based on the anterior corneal curvatures in the 1.0 to 4.0 mm central zone. A keratometric index of 1.3375 was used to calculate the powers of the steep and flat meridians. The posterior sim*K* was derived from the posterior axial curvature map as the arithmetic mean of the pair of orthogonal meridians, with the greatest difference in average power in the 0.5 to 2.0 mm zone. The refractive indices of the cornea (1.376) and aqueous humor (1.336) were used to calculate the powers of the steep and flat meridians.

### 3.2. Color Light-Emitting Diode Corneal Topographer

The Cassini v2.4.1 has approximately 700 red, yellow, and green LEDs arranged in a specific pattern to ensure a 1-to-1 correspondence between the source and image points, which potentially decreases source-image mismatch and artifacts caused by the shadow. The color-LED topographer evaluates the keratometric values in the 3.0 mm central zone. Additionally, the Cassini analyzes the reflections (2nd Purkinje images) of infrared LEDs on the posterior surface to calculate the posterior curvature. To calculate the anterior corneal surface astigmatism using the Cassini, we converted the anterior radii of curvature to meridional power using a keratometric index of 1.3375. Posterior corneal astigmatism was calculated using ray tracing, in which Snell's law is applied to calculate the refraction of a large number of light rays incident on the anterior and posterior corneal surface.

### 3.3. Statistical Analysis

Data analysis was performed using SPSS software (version 24.0, SPSS, Inc.) and Microsoft Office Excel (Microsoft Corp.). The results of the quantitative variables were expressed by their minimum and maximum values, means, and standard deviations (SDs). The mean anterior and posterior corneal powers were calculated for each measurement on each device as the arithmetic average of the anterior and posterior corneal steep *K* and flat *K*. The magnitude of corneal astigmatism was the measured difference between the steepest and flattest meridians, with its location along the steepest corneal meridian. Additionally, corneal astigmatism was expressed and compared using power vector analysis [8]. Each astigmatism value was converted to a Jackson (*J*) cross-cylinder notation, represented by the rectangular vectors *J*0 and *J*45, using the following equations:

(1) $$\begin{array}{c} J0\;=\left(\frac{C}{2}\right)\cos\left(2\emptyset \right),\\ J45=\;\left(\frac{C}{2}\right)\sin\left(2\emptyset \right), \end{array}$$

where *J*0 is the magnitude of a Jackson cross-cylinder with its axis at 0 degrees, *J*45 is the magnitude of a Jackson cross-cylinder with its axis at 45 degrees, *C* is the magnitude of the corneal astigmatism (the steepest *K* minus the flattest *K*), and ∅ is the axis of the steepest meridian [8].

To compare the measurements between the 2 devices, we performed paired sample *t-*tests. The ICC was calculated to analyze the repeatability and agreement of the results. A *p* value less than 0.05 was considered statistically significant, and ICC values higher than 0.900 were regarded as indicating a high degree of agreement. Bland–Altman plots, made with Stata software (version 9.2, Stata Corp. LP), were used to evaluate the agreement in corneal power and astigmatism between the dual rotating Scheimpflug–Placido and color-LED corneal topographers [9].

### 3.4. Sample Size Calculation

Based on the previous study, the standard deviation of the differences in corneal power measurements between devices was estimated to be 0.25*D* [10]. The smallest difference that may be clinically relevant was defined as 0.125*D*. With a significance level of 5% and a test power of 90%, at least 44 eyes were required in each group.

## 4. Results

The study enrolled 120 eyes from 120 subjects. Sixty eyes were in the normal group and 60 in the refractive group. There was no statistical difference in sex or age between the two groups. As expected, refractive errors were statistically significantly smaller in the refractive group (Table 2).

### 4.1. Repeatability

The repeatability of both the dual rotating Scheimpflug–Placido system and the color-LED corneal topographer was excellent for anterior sim*K* (ICC = 0.998 and ICC = 0.992, respectively), anterior astigmatism magnitude (ICC = 0.980 and ICC = 0.984, respectively), anterior *J*0 (ICC = 0.991 and ICC = 0.988, respectively), anterior *J*45 (ICC = 0.993 and ICC = 0.996, respectively), posterior sim*K* (ICC = 0.985 and ICC = 0.983, respectively), posterior astigmatism magnitude (ICC = 0.971 and ICC = 0.973, respectively), posterior *J*0 (ICC = 0.969 and ICC = 0.976, respectively), and posterior *J*45 (ICC = 0.977 and ICC = 0.972, respectively).

### 4.2. Anterior Corneal Indices

Tables 3 and 4 compare the measurements of the anterior corneal indices between the dual rotating Scheimpflug–Placido and color-LED corneal topographers. The measurements for anterior sim*K*, anterior astigmatism magnitude, anterior *J*0, and anterior *J*45 did not differ significantly between the two devices in either group (*p* > 0.05), with the exception of anterior sim*K* in the normal group (*p*=0.001) and anterior *J*45 in the postrefractive group (*p*=0.000). The ICC values between the two devices were high for anterior sim*K*, anterior astigmatism magnitude, anterior *J*0, and anterior *J*45 in both the groups (ICC > 0.900).

### 4.3. Posterior Corneal Indices

**Figure 1:**
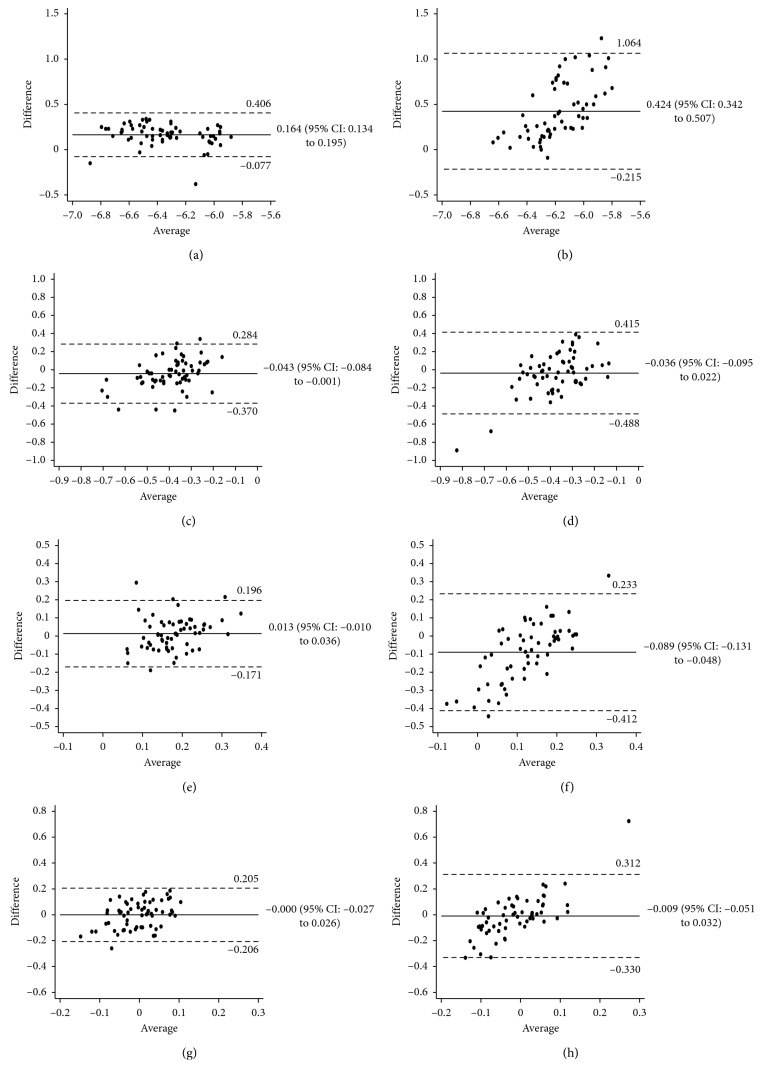
Bland–Altman plots showing the agreement between the posterior corneal index measurements from the 2 systems. The bold lines represent the mean difference between the 2 methods. The broken lines represent the 95% limits of agreement. (a) Normal group posterior sim*K*, (b) refractive group posterior sim*K*, (c) normal group posterior astigmatism magnitude, (d) refractive group posterior astigmatism magnitude, (e) normal group posterior *J*0, (f) refractive group posterior *J*0, (g) normal group posterior *J*45, and (h) refractive group posterior *J*45 (color LED, color light-emitting diode corneal topographer; DRSP, dual rotating Scheimpflug–Placido corneal topographer).

Tables 3 and 4 also compare the measurements of the posterior corneal indices between the two instruments. The measurements for posterior sim*K* and posterior astigmatism magnitude in the normal group and posterior sim*K* and posterior *J*0 in the postrefractive group differed significantly between the 2 devices (*p* < 0.05). The values for posterior *J*0 (*p*=0.274) and posterior *J*45 (*p*=0.977) in the normal group and posterior astigmatism magnitude (*p*=0.216) and posterior *J*45 (*p*=0.655) in the postrefractive group did not differ significantly. The ICC values between the two devices were low for posterior sim*K*, posterior astigmatism magnitude, posterior *J*0, and posterior *J*45 in both the groups (ICC < 0.900), with the exception of posterior sim*K* in the normal group (ICC = 0.944). Bland–Altman plots showed poor agreement between the devices in the posterior corneal indices (Figure 1).

## 5. Discussion

The cornea is one of the most important refractive elements of the eye; thus, a precise evaluation of its characteristics is mandatory and requires the use of reliable measurement devices. Generally, it is essential to compare and evaluate new measurement instruments to determine whether there is an adequate agreement among different modalities [9]. Estimating and comparing the repeatability of different instruments is basic research for data reliability and determination of a better system. An agreement in measurements assesses the exchangeability of devices and serves as an indirect indicator of accuracy [11]. This study was designed to evaluate the repeatability and agreement of anterior and posterior corneal power and astigmatism measurements acquired from a dual rotating Scheimpflug–Placido topographer and a color-LED topographer.

Traditionally, the total corneal power calculation is based on the anterior corneal surface measurements, assuming a fixed anterior and posterior curvature ratio to estimate the posterior corneal power [12]. The standardized keratometric index (1.3375 for most cases) has been used when converting anterior corneal measurements into total corneal power and astigmatism. However, this reasoning has reportedly led to errors [13, 14]. Although diverse technologies, such as Scheimpflug imaging, slit-scanning technology, and optical coherence tomography, enable measurement of the posterior corneal surface, various levels of repeatability and agreement among measurement systems have been reported; therefore, the need to develop more accurate instruments remains high. To our knowledge, this is the first study to report the comparability and repeatability of a recently introduced device, the Cassini point-source color-LED topographer, for posterior corneal assessment.

Previous studies have reported various repeatability outcomes for the corneal power and astigmatism measurements from the dual rotating Scheimpflug–Placido system (Galilei G4) and the Cassini color-LED corneal topographer. Ventura et al. found that the ICCs for corneal power from the color-LED topographer, the Placido topographer, and a reflectometer were all greater than 0.960, although the ICC for the color-LED topographer was the lowest [15]. And they also found that while the astigmatism measurements from the 3 devices were all highly repeatable, the color-LED topographer had statistically lower repeatability than the others [15]. Klijn et al. reported that the repeatability of the Cassini corneal power measurements was not statistically different from that of the Keratron, but it was lower than those of the Lenstar and Pentacam, suggesting that the discrepancy might result from Cassini's high sensitivity to misalignment of the cornea [16]. And they also demonstrated that Cassini's repeatability for cylinder measurements was significantly higher than those of the Keratron and Pentacam [16]. However, in this study, the dual rotating Scheimpflug–Placido and color-LED corneal topographers both provided highly repeatable corneal power and astigmatism measurements, achieving ICCs of greater than 0.968 and 0.952 in the normal and postrefractive groups, respectively. The previous studies were performed using an earlier version of the Cassini, which could explain the differences to our results.

The anterior corneal power and astigmatism measurements provided by the color-LED corneal topographer did not differ significantly from those of the dual rotating Scheimpflug–Placido system in either the normal or postrefractive group, with the exception of the anterior sim*K* in the normal group and the anterior *J*45 in the postrefractive group. The anterior corneal sim*K* measurement was significantly higher with the Cassini topographer than with the Galilei topographer, with a mean difference of 0.12*D*. However, that value is smaller than the diurnal cornea variation [17] or the minimum measurement scale. Furthermore, as there is no sufficient standard reference for keratometry measurements, it is difficult to determine the accuracy of the devices [9, 18]. Therefore, we evaluated and compared the quantitative agreement by calculating the ICC between the two devices [18]. Agreement was high for all the anterior corneal power and astigmatism measurements, with ICCs greater than 0.905 in both the normal and postrefractive groups. Previously, Ventura et al. reported no statistically significant differences in corneal power measurements in normal or postrefractive surgery eyes or astigmatism in postrefractive surgery eyes between the color-LED device and the Placido or dual-Scheimpflug devices [15], which accords with our study.

In this study, we found statistically significant differences in all posterior parameters except for the posterior *J*45 of the total group (120 eyes with and without a history of refractive surgery), the posterior sim*K* and posterior astigmatism magnitude of the normal group, and the posterior sim*K* and posterior *J*0 of the postrefractive group. Furthermore, agreement ICCs were lower than 0.681 in all parameters for the total group and the subgroups, except only the posterior sim*K* of the normal group (ICC = 0.944). But more specifically, as shown in Figure 1, there was a greater variability in the group of patients who underwent refractive surgery. Previous studies also reported greater differences of the estimated values in the postrefractive group than in the normal group, because refractive surgeries change the corneal centration and eccentricity and assumed the ratio of anterior-to-posterior radius of the curvature. This may potentially have an influence on the estimated values of the two devices.

The first explanation for the low ICCs in the posterior parameters is the differences in the measurement principles. The dual rotating Scheimpflug–Placido topographer derives the mean posterior corneal power from the Scheimpflug data [19], whereas the color-LED topographer analyzes the reflections of 7 infrared LEDs on the posterior surface. And the measurement zone of the Galilei topographer is from 0.5 to 2.0 mm from the center, while the Cassini topographer evaluates keratometry values in the 3.0 mm central zone. Furthermore, the acquisition time with the Cassini (v2.4.1) is about 2 seconds with 20 instantaneous frame acquisitions; the Galilei, in contrast, requires 0.75 seconds. Within our knowledge, more recent version of the Cassini (v2.5) is expected to have shorter acquisition time, since it only needs 3 individual instantaneous acquisitions. As there is no reference system to confirm which system comes closer to the real values, we cannot conclude which system provides the correct values of the posterior cornea.

A major limitation of this study was that the keratometry measurement zones for the Cassini and Galilei are different. Different optical zones of the two different devices made it impossible for us to compare values exactly under the same conditions; however, as a characteristic of the comparative study, this difference of the result should be noticed and appreciated as well. Nevertheless, we unified all configurable settings as the keratometric index to ensure the comparability of corneal parameters. Also, most studies, including this one, enroll young, healthy patients who can cooperate well with the tests; however, older patients with poorer cooperation can produce different outcomes. Moreover, a larger number of participants are needed to clarify the various tendencies of each parameter. Finally, the clinical relevance of this study, such as postoperative results, warrants further studies.

## 6. Conclusion

This study is the first to compare both anterior and posterior keratometries between the dual rotating Scheimpflug–Placido and color-LED corneal topographers. The color-LED corneal topographer and dual rotating Scheimpflug–Placido topographer showed high agreement for anterior corneal measurements; however, the agreement was low for posterior corneal indices. These results were similar in both the normal and the postrefractive groups.

## Figures and Tables

**Table 1:** Characteristics and measurement settings for each system.

Parameter	Dual rotating Scheimpflug–Placido	Color light-emitting diode
Acquisition time (s)	0.75	2
*K* index	1.3375^*∗*^	1.3375^*∗*^
*K* measurement zone (mm)	1–4 (anterior)	3
	0.5–2 (posterior)	

*K*, keratometry. ^*∗*^Configurable or selectable option.

**Table 2:** Demographics and refractive error by group.

Parameter	Refractive group (*n* = 60)	Normal group (*n* = 60)	*p* value
Mean age (y) ± SD	32.30 ± 8.05	32.78 ± 6.98	0.684^*∗*^
Sex, *n* (%)			0.133^†^
** **Male	24 (40.0)	32 (53.3)	
** **Female	36 (60.0)	28 (46.7)	
Mean SE (*D*) ± SD (range)	−0.42 ± 0.46 (−1.75–0.50)	−2.28 ± 2.25 (−5.50–0.75)	<0.001^∗^
Surgery, *n* (%)			
** **LASEK	48 (80.0)	─	
** **LASIK	12 (20.0)	─	

LASEK, laser-assisted subepithelial keratectomy; LASIK, laser in situ keratomileusis; SE, spherical equivalent. ^*∗*^Independent sample *t*-test; ^†^chi-square test.

**Table 3:** Comparison of corneal index measurements using dual rotating Scheimpflug–Placido and color light-emitting diode corneal topographers.

Parameter	DRSP (mean ± SD)	Color LED (mean ± SD)	Difference		*p* value^*∗*^	ICC
			Mean ± SD	95% CI		
Postrefractive group						
*Anterior corneal indices* (*D*)						
sim*K*	38.78 ± 1.80	38.71 ± 1.88	−0.08 ± 0.44	−0.19, 0.04	0.186	0.986
Astigmatism magnitude	0.83 ± 0.47	0.84 ± 0.53	0.01 ± 0.37	−0.09, 0.10	0.904	0.938
*J*0	−0.32 ± 0.28	−0.29 ± 0.32	0.03 ± 0.20	−0.02, 0.08	0.267	0.925
*J*45	0.06 ± 0.22	−0.04 ± 0.25	−0.10 ± 0.15	−0.14, −0.06	0.000	0.905
*Posterior corneal indices* (*D*)						
sim*K*	−6.39 ± 0.16	−5.97 ± 0.32	0.42 ± 0.32	0.34, 0.51	0.000	0.327
Astigmatism magnitude	−0.36 ± 0.11	−0.39 ± 0.21	−0.04 ± 0.23	−0.09, 0.02	0.216	0.183
*J*0	0.17 ± 0.06	0.08 ± 0.16	−0.09 ± 0.16	−0.13, −0.05	0.000	0.016
*J*45	−0.02 ± 0.15	−0.01 ± 0.06	−0.01 ± 0.16	−0.05, 0.03	0.655	0.000
Normal group						
*Anterior corneal indices* (*D*)						
sim*K*	43.28 ± 1.37	43.40 ± 1.38	0.12 ± 0.26	0.06, 0.19	0.001	0.991
Astigmatism magnitude	1.34 ± 0.83	1.41 ± 0.88	0.07 ± 0.35	−0.02, 0.15	0.133	0.956
*J*0	−0.61 ± 0.44	−0.64 ± 0.47	−0.03 ± 0.17	−0.08, 0.01	0.135	0.963
*J*45	0.01 ± 0.25	0.00 ± 0.24	−0.01 ± 0.14	−0.04, 0.03	0.740	0.916
*Posterior corneal indices* (*D*)						
sim*K*	−6.43 ± 0.27	−6.26 ± 0.25	0.16 ± 0.12	0.13, 0.19	0.000	0.944
Astigmatism magnitude	−0.37 ± 0.11	−0.41 ± 0.17	−0.04 ± 0.16	−0.08, −0.00	0.043	0.499
*J*0	0.17 ± 0.07	0.18 ± 0.09	0.01 ± 0.09	−0.01, 0.04	0.274	0.469
*J*45	0.00 ± 0.06	0.00 ± 0.09	−0.00 ± 0.10	−0.03, 0.03	0.977	0.175

CI, confidence interval; color LED, color light-emitting diode corneal topographer; DRSP, dual rotating Scheimpflug–Placido corneal topographer; ICC, intraclass correlation coefficient; K, keratometry. ^*∗*^Paired sample *t*-test.

**Table 4:** Comparison of corneal index measurements using dual rotating Scheimpflug–Placido and color light-emitting diode corneal topographers in total group.

Parameter	Total group					
	DRSP (mean ± SD)	Color LED (mean ± SD)	Difference		*p* value^*∗*^	ICC
			Mean ± SD	95% CI		
*Anterior corneal indices* (*D*)						
sim*K*	41.07 ± 2.76	41.09 ± 2.87	0.03 ± 0.37	-0.04, 0.09	0.451	0.996
Astigmatism magnitude	1.09 ± 0.72	1.13 ± 0.78	0.04 ± 0.36	−0.03, 0.10	0.256	0.938
*J*0	−0.46 ± 0.39	−0.47 ± 0.44	−0.00 ± 0.19	−0.04, 0.03	0.874	0.947
*J*45	0.04 ± 0.24	−0.02 ± 0.25	−0.05 ± 0.15	−0.08, 0.03	0.000	0.906
*Posterior corneal indices* (*D*)						
sim*K*	−6.41 ± 0.22	−6.12 ± 0.32	0.29 ± 0.27	0.24, 0.34	0.000	0.681
Astigmatism magnitude	−0.36 ± 0.11	−0.40 ± 0.19	−0.04 ± 0.20	−0.07, −0.00	0.027	0.334
*J*0	0.17 ± 0.06	0.13 ± 0.13	−0.04 ± 0.14	−0.06, −0.01	0.004	0.180
*J*45	−0.01 ± 0.06	−0.01 ± 0.12	−0.00 ± 0.13	−0.03, 0.02	0.695	0.000

CI, confidence interval; color LED, color light-emitting diode corneal topographer; DRSP, dual rotating Scheimpflug–Placido corneal topographer; ICC, intraclass correlation coefficient; K, keratometry. ^*∗*^Paired sample *t*-test.

## Data Availability

The data used to support the findings of this study are available from the corresponding author upon request.

## List of abbreviations

ICC: Intraclass correlation coefficient
LED: Light-emitting diode
*D*: Diopters
I-S: Inferior-superior
sim*K*: Simulated *K*
SD: Standard deviations
J: Jackson
LASEK: Laser-assisted subepithelial keratectomy
LASIK: Laser in situ keratomileusis
SE: Spherical equivalent
CI: Confidence interval
Color LED: Color light-emitting diode corneal topographer
DRSP: Dual rotating Scheimpflug–Placido corneal topographer
K: Keratometry.
